# A targeted approach with nanopore sequencing for the universal detection and identification of flaviviruses

**DOI:** 10.1038/s41598-021-98013-9

**Published:** 2021-09-24

**Authors:** Patrick Reteng, Linh Nguyen Thuy, Tam Tran Thi Minh, Maria Angélica Monteiro de Mello Mares-Guia, Maria Celeste Torres, Ana Maria Bispo de Filippis, Yasuko Orba, Shintaro Kobayashi, Kyoko Hayashida, Hirofumi Sawa, William W. Hall, Lan Anh Nguyen Thi, Junya Yamagishi

**Affiliations:** 1 Division of Collaboration and Education, International Institute for Zoonosis Control, Hokkaido University, Sapporo, Japan; 2 Center for Bio-Medical Research, National Institute of Hygiene and Epidemiology, Hanoi, Vietnam; 3 Flavivirus Laboratory, Oswaldo Cruz Institute, Fiocruz, Rio de Janeiro, Brazil; 4 Division of Molecular Pathobiology, International Institute for Zoonosis Control, Hokkaido University, Sapporo, Japan; 5 Laboratory of Public Health, Faculty of Veterinary Medicine, Hokkaido University, Sapporo, Japan; 6 International Collaboration Unit, International Institute for Zoonosis Control, Hokkaido University, Sapporo, Japan; 7 Global Virus Network, Baltimore, USA; 8 National Virus Reference Laboratory, University College Dublin, Dublin, Ireland; 9 Ireland Vietnam Blood-Borne Virus Initiative (IVVI), Dublin, Ireland

**Keywords:** Infectious diseases, Next-generation sequencing

## Abstract

Nucleic acid test (NAT), most typically quantitative PCR, is one of the standard methods for species specific flavivirus diagnosis. Semi-comprehensive NATs such as pan-flavivirus PCR which covers genus *Flavivirus* are also available; however, further specification by sequencing is required for species level differentiation. In this study, a semi-comprehensive detection system that allows species differentiation of flaviviruses was developed by integration of the pan-flavivirus PCR and Nanopore sequencing. In addition, a multiplexing method was established by adding index sequences through the PCR with a streamlined bioinformatics pipeline. This enables defining cut-off values for observed read counts. In the laboratory setting, this approach allowed the detection of up to nine different flaviviruses. Using clinical samples collected in Vietnam and Brazil, seven different flaviviruses were also detected. When compared to a commercial NAT, the sensitivity and specificity of our system were 66.7% and 95.4%, respectively. Conversely, when compared to our system, the sensitivity and specificity of the commercial NAT were 57.1% and 96.9%, respectively. In addition, Nanopore sequencing detected more positive samples (n = 8) compared to the commercial NAT (n = 6). Collectively, our study has established a semi-comprehensive sequencing-based diagnostic system for the detection of flaviviruses at extremely affordable costs, considerable sensitivity, and only requires simple experimental methods.

## Introduction

Members of the genus *Flavivirus* (family Flaviviridae) include important pathogens such as yellow fever virus (YFV), Japanese encephalitis virus (JEV), St. Louis encephalitis virus (SLEV), Dengue virus (DENV), West Nile virus (WNV), Tick borne encephalitis virus (TBEV), and Zika virus (ZIKV). These viruses pose threats to public health globally, as multiple outbreaks caused by these viruses have occurred^1^. Diagnosing flavivirus infection during the acute phase can be challenging due to non-specific clinical manifestations, cross-reactivity of serologic methods used for flavivirus detection, and undetectable antibody titres during acute phase^2–4^. Antigen tests are available for dengue detection, but the test has limitations in differentiating the different serotypes of DENV. Nucleic acid tests (NATs), such as conventional polymerase chain reaction (PCR) and quantitative polymerase chain reaction (qPCR) have gained more attention for confirmation of flaviviruses infection^5,6^. These tests are pathogen specific; however, a presumption of target pathogen is required, and this can lead to an oversight of other pathogens which might also be present.

Comprehensive approaches targeting 16S rRNA and 18S rRNA are effective for prokaryote and eukaryote pathogen detection, respectively. However, the approaches are not feasible for virus detection due to the lack of conserved sequences which can cover whole virus species. In contrast, conserved sequences can be found in viruses at certain family or genus levels and by targeting those sequences, semi-comprehensive detection approaches at family or genus level can be achieved. For flaviviruses, a semi-comprehensive detection system was developed by PCR targeting a conserved region that encodes non-structural protein 5 (NS5)^7^. This provides a rapid detection platform, but with limitations on its specificity. For species differentiation, additional analysis such as detailed sequencing or the employment of less feasible TaqMan probes, are required.

Oxford Nanopore is a cost-effective and portable sequencing platform. It also minimizes the laboratory-oriented process, allowing sequencing to be performed even in the field^8,9^. This sequencing platform can also provide real time data, ideal for diagnostic purposes. As a trade-off for its size and portability, the Nanopore sequencing platform lacks the sequencing accuracy when compared to other next generation sequencing platforms (5–15% raw sequencing error rate)^10^. This error rate is of concern in analysis where high-quality data is needed (genome assembly, single nucleotide polymorphism) but can be accommodated for pathogen detection with pipelines to minimize the errors and plausible read-count threshold. Hence, we integrated the pan-flavivirus PCR and Nanopore sequencing to develop a semi-comprehensive detection system for the genus flavivirus as a model of sequencing-based diagnosis, namely diagnosis-by-sequencing. As a series of proof-of-concept studies, we validated the system using both in vitro-prepared spiked samples and clinical samples.

## Results

### Modification of pan-flavivirus primers

**Figure 1:**
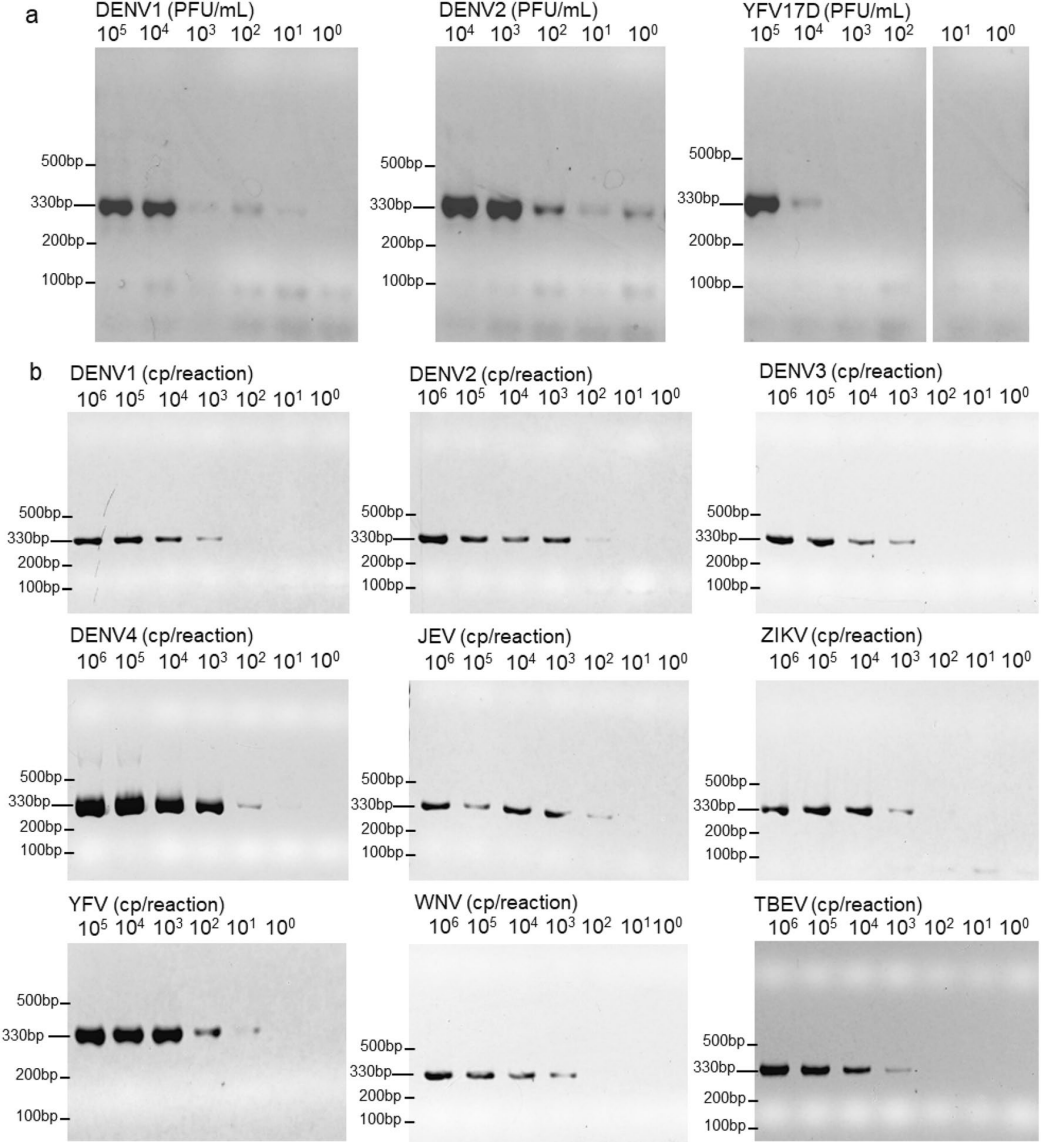
Sensitivity of PCR using the modified pan-flavivirus primers. (**A**) Fetal bovine serum were spiked with DENV1, DENV2, and YFV viral particles. RNAs were extracted from them then used as PCR template. (**B**) Plasmids containing the indicated sequences were used. Images were cropped from the original. Original image can be found in Supplementary Fig. S3 and S4. *DENV* Dengue Virus, *JEV* Japanese Encephalitis Virus, *ZIKV* Zika Virus, *YFV* Yellow Fever Virus, *WNV* West Nile Virus, *TBEV* Tick Borne Encephalitis Virus.

The primer set targeting the conserved region in the flavivirus NS5 gene^7^ was modified to achieve multiplex sequencing. Twenty-four unique nucleotide sequences (index) were generated with Free Right End Edit Barcodes (FREEBarcodes)^11^ and were concatenated to the 5’ end of the primers. The sense and anti-sense primers were given a different set of indexes, resulting in 12 sets of indexed primer that could be used to multiplex up to 144 samples (Supplementary Table S1). The modified primers were shown to successfully amplify the target sequences from DENV1, DENV2, and YFV17D from spiked samples up to 10 PFU/mL, 1 PFU/mL, and 10^4^ PFU/mL, respectively (Fig. 1a).

### Optimization of bioinformatic analysis

RNA extracted from virus-spiked samples containing 10^5^ PFU/mL (DENV1, YFV17D) or 10^4^ PFU/mL (DENV2) were subjected to pan-flavivirus reverse-transcriptase polymerase chain reaction (RT-PCR) with the indexed primer and then sequenced according to the workflow (Supplementary Fig. S1 and the data will be shown below after the section of parameter optimization). In triplicate, twelve samples with different combinations of virus, index and barcode were sequenced. After the analysis, the barcode, index, and viral sequence were obtained for each read (Supplementary Table S3). Their combination was known according to the sample; therefore, we could identify the origin of the reads (sample ID) if all of the three properties were consistent with the known combination. On the other hand, if two out of the three properties were corresponded to the known combination, we could still identify the origin of the read. Simultaneously, we could identify miss-assigned properties which would be used for error ratio estimation (Supplementary Fig. S1b). In contrast, if all of the three were inconsistent, we could not obtain any reliable information from the reads (Supplementary Fig. S1).

Three different bioinformatics approaches were used and compared for the deindexing: FREEBarcodes, Local Alignment Search Tool (LAST) v 916^12^, and Minibar v 0.2.1^13^. Different parameters for each tool were tested and compared. Sequence data obtained from spiked samples showed that the overall recovery rate (percentage of deindexed reads to debarcoded reads) was less than 50% in any of the three tools (Supplementary Table S2). Among the three tools, FREEBarcodes yielded the lowest false results in index rate but also had the lowest recovery rate. In contrast, both LAST and Minibar pipeline showed a better read-recovery rate, but Minibar suffered from high false results in index rate ranging from 1.11 to 3.10% (Supplementary Table S2). Deindexing with LAST (alignment score 70) was adopted because it showed extremely limited false results in index rate (0.01% to 0.03%; average 0.02%), despite not having the highest recovery rate. The comparison also showed that alignment score of 70 for LAST generate a balance of recovery rate and false in index rate. Using this pipeline and parameters, this system can correctly identify the virus spiked in the samples; either using MinION or Flongle (Table 1, Supplementary Table S3).

**Table 1:** Details of the reads obtained from validation of the system using spiked sample and the optimized pipeline.

Virus in the sample	MinION							Flongle 1							Flongle 2						
	Debarcoded reads^a^	Deindexed reads^b^	Viral reads					Debarcoded reads^a^	Deindexed reads^b^	Viral reads					Debarcoded reads^a^	Deindexed reads^b^	Viral reads				
			YFV	DENV1	DENV2	Other Flaviviruses	Other Virus			YFV	DENV1	DENV2	Other Flaviviruses	Other Virus			YFV	DENV1	DENV2	Other Flaviviruses	Other Virus
DENV1	2960	684	0	607	0	0	0	1669	447	0	408	0	0	0	6632	2291	0	1872	0	0	0
DENV2	5062	1454	0	0	1055	2	0	1254	182	0	0	151	0	0	5243	1711	0	0	1187	0	0
YFV	3066	397	350	0	0	0	0	964	362	329	0	0	0	0	3970	1085	943	3	4	0	0
DENV1	5312	2068	0	1784	0	0	0	1237	312	0	276	0	0	0	5563	709	0	573	0	0	0
DENV2	2541	271	0	0	203	0	0	676	483	0	0	400	1	0	2384	737	0	0	515	1	0
YFV	5942	1988	1754	0	0	0	0	1184	272	254	0	0	0	0	687	155	134	0	0	0	0
DENV1	1583	306	0	266	0	0	0	607	210	0	190	0	0	0	4163	1666	0	1388	0	0	0
DENV2	1796	498	0	0	353	0	0	2304	91	0	0	70	0	0	8422	3254	0	1	2251	2	0
YFV	1978	506	457	0	0	0	0	1431	371	338	0	0	0	0	3630	845	751	0	0	0	0
DENV1	2382	541	0	492	0	0	0	993	459	0	401	0	0	0	5323	1790	0	1555	0	0	0
DENV2	4183	533	0	0	410	2	0	1051	809	0	0	618	4	0	7288	2045	0	1	1400	2	0
YFV	3253	456	403	0	0	0	0	1010	273	243	0	0	0	0	2613	791	**657**	0	3	0	0

Viral RNA was extracted from fetal bovine serum spiked with 10^5^ PFU/mL DENV1, 10^4^ PFU/mL DENV2, and 10^5^ PFU/mL YFV17D, then subjected to RT-PCR and sequencing. The number of reads shown in the table were obtained the optimized pipeline data was analysed according to the pipeline shown in the Supplementary Fig. 1

^a^Reads survived after the debarcoded step is referred to debarcoded reads.

^b^While reads passing the deindexing step will be referred as deindexed reads.

Despite the above optimization, the false results in index rate was unable to reach zero. This will make it difficult to discriminate true reads from artifact derived from read migration. False index reads are supposedly to be mostly cross-talk which originated from the neighbouring samples with identical index in either forward or reverse index. Therefore, an approach was developed to determine the read threshold based on Poisson distribution, given the rate (λ) derived from the observed ratio of the false in index and the number of reads from the neighbouring samples (Supplementary Fig. S2). Particularly for indexes used in this study, adopted sequencing system, and bioinformatics pipeline, the error ratio was 0.02%. This ratio was multiplied by 10 as a safety margin. For example, in one of the in vitro validation study, there were 1,055 DENV2 reads in sample i02-i14 and one DENV2 reads in sample i07-i14 (Supplementary Table S3, Supplementary Fig. S2). These reads share the same reverse index (i14), thus the assumption is that the i07-i14 read is a cross-talk, originated from i02-i14 reads, as a result of errors that causes index i02 to be classified as index i07. We hypothesized that the errors occur in stochastic manner following Poisson distribution with the rate (λ) derived from number of reads and error ratio. In this case, they are 1,056 and 0.02%, respectively, which leads to the sample with number of reads between one to six are likely to be cross-talk (P ≥ 0.01) (Supplementary Fig. S2).

### Validation of employing flaviviruses, with sensitivity, and reproducibility

Once the RT-PCR and sequencing system had been established, a range in flaviviruses were examined. The target sequences from nine flaviviruses were cloned into plasmids and used as templates. On gel electrophoresis, amplicons were clearly observed at 10^3^ copies (DENV1, DENV2, DENV3, ZIKV, TBEV, YFV), and 10^2^ copies (DENV4, JEV) of template per reaction (Fig. 1b). While, in PCR reactions using original index-free primers, amplicons were visible in reactions with 10 to 10^2^ copies of template per reaction lower compared to when using indexed primers (Supplementary Fig. S4), suggesting reduced efficacy in PCR by the 26-nt additional index sequences.

The amplicons obtained from PCR with indexed primers were sequenced with Flongle resulted in 244,851 raw reads, 53,894 debarcoded reads, and 16,366 deindexed reads (Supplementary Table S4). Alignment search results showed that the system can differentiate all nine flaviviruses in the sample. Similar experiments were carried out for the faint or sub-visible amplicons using the MiniION flowcell in which a substantial number of DENV3 and DENV4 reads were able to be detected among 3,377 raw reads (Supplementary Table S5). Collectively, reads could be detected at least from 10^3^ copies (DENV1, DENV2, JEV, TBEV, WNV, YFV, and ZIKV), 10^2^ copies (DENV3), and 10^1^ copies (DENV4) of template per reaction.

The reproducibility of the system was also evaluated. Experiments using FBS spiked with viral particles were performed in triplicate as mentioned above (Table 1, Supplementary Table S3). In all experiments, our pipeline consistently identified the virus species which was spiked in the sample. In addition, the other parameters (such as recovery rate and false results in index rate) showed consistent results across the experiments (Supplementary Table S2).

### System validation using clinical samples

For the 114 clinical samples from Vietnam, sequencing with Flongle yielded 3,126 deindexed reads. Meanwhile, deep sequencing of the samples with invisible amplicons using MinION yielded 80,579 deindexed reads (Supplementary Tables S6 and S7). Collectively, three DENV serotypes were detected in this test: DENV1, DENV2, and DENV4 (Table 2, Supplementary Tables S8 and S9). Three samples (i01-i20, i06-i19, and i06-i23) were positive by gel-electrophoresis (Supplementary Fig. S5) and all the three were successfully sequenced by Flongle. When deep sequencing was conducted with the MinION, Dengue virus sequences were obtained from additional five samples (i04-i15, i06-i15, i06-i20, i06-i22, and i08-i20) which were invisible by gel-electrophoresis (Supplementary Fig. S5). Interestingly, one of them (i08-i20) was obtained from a NS1 antigen-test negative sample collected one day after fever onset (Table 2). Meanwhile, 20 and 12 samples with limited numbers of DENV reads by Flongle and MinION, respectively, were regarded as negative because the numbers were below their threshold. For example, when the samples were sequenced by Flongle, three DENV1 reads were acquired from the i01-i19 indexed sample, but this sample was considered negative when subjected to our calculation (λ = 0.48, P(x ≥ 3) = 0.013, Supplementary Fig. S2 and Supplementary Table S8). While in the results of the MinION sequencing, a sample with the i10- i13 index pair showed a very limited number of reads yet it is still above the threshold. This sample will be regarded as false positive as well, due to limited read number (≤ 2 reads). In comparison to the NS1 test, the sensitivity and specificity of the sequencing test using Flongle were 4.23% and 100%, respectively and those with the MinION-integrated Nanopore (Flongle and MinION) tests were 9.86% and 97.67%. In contrast, in comparison to a dengue specific reverse transcriptase qPCR (RT-qPCR) test, the sensitivity and specificity of the sequencing test using Flongle were 33.33% and 98.46%, respectively. Those of the MinION-integrated test were 66.67% and 95.38%. On the other hand, when compared to sequencing test using Flongle, the sensitivity and specificity of the RT-qPCR were 66.67% and 94.12% respectively and when it is compared to MinION-integrated test, the sensitivity and specificity were 57.14% and 96.88%, respectively (Table 3).

**Table 2:** Data of the positive results for RT-PCR and sequencing obtained from clinical samples in Vietnam.

Index	Day of fever	NS1^a^	^b^Ct Value (qPCR)	RT-PCR^c^	Flongle	Minion	^d^Number of reads					
							DENV1	DENV2	DENV3	DENV4	Other flaviviruses	Other viruses
i01-i20	4	+	−	+	DENV2	Not tested	3	44	0	0	0	0
i03-i15	6	+	−	−	−	DENV1	12	0	0	0	0	0
i06-i15	2	+	−	−	−	DENV4	0	0	0	16	0	0
i06-i18	3	+	39	−	−	−	0	0	0	0	0	0
i06-i19	1	+	28.2	+	DENV1	Not tested	179	0	0	0	0	0
i06-i20	4	+	41.5	−	−	DENV1	187	0	0	0	0	0
i06-i21	2	+	35.8	−	−	−	0	0	0	0	0	0
i06-i22	3	+	34	−	−	DENV1	107	0	0	0	0	0
i06-i23	2	+	32.5	+	DENV1	Not tested	43	0	0	0	0	0
i08-i20	1	−	Not tested	−		DENV1	60	0	0	0	0	0

Viral RNA was extracted from 114 serum sample, then subjected to pan-flavivirus RT-PCR and sequencing. First-round of sequencing was carried out using a Flongle flowcell. Positive samples were then excluded from the second-round sequencing (using a MinION flowcell).

^a^As a comparison, the samples were also screened with the NS1 antigen test.

^b^Samples positive for NS1 were also subjected to the CDC based DENV-1-4 Real-Time RT-PCR Multiplex Assay.

^c^Prior to sequencing, 1 µL of PCR product were visualized on agarose, alongside 100 bp DNA ladder (Supplementary Fig. S5).

^d^Reads meeting the criteria were counted at the end of analysis and presented here.

**Table 3:** Sensitivity and specificity of clinical result.

Comparative assay	Test	Sensitivity (positive agreement) (%)	Specificity (negative agreement) (%)
NS1	gel	4.23	100.00
NS1	Flongle	4.23	100.00
NS1	Nanopore	9.86	97.67
RT-qPCR	Flongle	33.33	98.46
RT-qPCR	Nanopore	66.67	95.38
Flongle	RT-qPCR	66.67	94.12
Nanopore	RT-qPCR	57.14	96.88

For the 24 clinical samples from Brazil, the pan-flavivirus RT-PCR was also conducted using a similar dual index approach, but with shorter, 14-nt index sequences (Supplementary Table S1B). This shorter index was a prototype of the indexing system, before we further modified it to 26-nt index used in Vietnam samples. On gel electrophoresis, clearly visible amplicons were obtained from 12 samples. On the other hand, there were five ambiguous samples and seven negative samples. These PCR products, including the ambiguous and negative samples, were sequenced with MinION and yielded 2,813,043 raw reads and deindexing with LAST resulted in 1,082,361 deindexed reads. Then sequences homologous to SLEV, YFV, WNV, DENV, and ZIKV were identified (Supplementary Table S10). Detection of flavivirus by RT-qPCR and the pan-flavivirus-MinION system was mostly comparable for the positive amplicons; however, several discrepancies were observed for the ambiguous and negatives samples. From this data, we were able to demonstrate that the system can detect a broad-spectrum of flavivirus in clinical samples.

## Discussion

In this study, a pan-flavivirus RT-PCR was combined with Nanopore sequencing to provide a broad flavivirus detection and identification platform. The system includes a multiplexing strategy with dual index primers. With a series of validations, the Nanopore system was shown to be able to detect a range of flaviviruses, with a comparable performance to a commercial NAT.

During the course of disease, reported Dengue virus loads in blood during infection ranged from 10^2^ to 10^5^ PFU/mL^14–16^. On electrophoresis, amplifications were observed from samples containing up to 1 or 10 PFU/mL which were in the range of clinical infections (Fig. 1b). The viral load during other flaviviruses infection range from 7.5 × 10^2^ to 5 × 10^5^ RNA copies (cp)/mL^17–20^. During validation with plasmids, amplifications were observed up to 10^2^ or 10^3^ cp per reaction (corresponding to approximately 2 × 10^4^ to 2 × 10^5^ viral genome cp/mL input of the pan-flavivirus-MinION system) which is still in the range of flavivirus viral loads during clinical disease (Fig. 1b). This suggests that the system is promising for social implementation as a point-of-care diagnosis. The unmodified pan-flavivirus primer as reported^7^ was able to detect 10–100 copies lower than the modified primer, indicating that the extension of index sequence may inhibits the PCR reaction. The long index sequence might affect the primer’s annealing to the template and/or inhibit reaction through primer dimer formation as observed in the gel electrophoresis image especially for the samples with lower template concentration (Supplementary Figs. S3 and S4). The main reason for the long index sequence is to minimize index migration due to sequencing error that is common in the Nanopore sequencer. However, the sequencing platform has received a major improvement in accuracy with the new R10.3 flowcell in pair with new basecalling tool (Bonito)^21^. The accuracy has been validated in several studies showing improvements in both consensus^22^ and sequencing accuracy^23^, including sequencing accuracy for the homopolymer region^22,24^. With the improved accuracy, index sequences can be shortened to minimize dimer formation, which should improve PCR efficiency.

Through the multiplex sequencing, we have detected limited but substantial number of reads from unassigned index sets (Supplementary Tables S6–S9). They were possible migrations from other reads as a result of sequencing error at one of the index sequences. Reads with errors at both sides can be ignored because the error ratio is squared. To set the threshold which differentiate true results from false results in index, a model based on Poisson distributions was applied (Supplementary Fig. S2). In this study, we applied Poisson distribution because number of obtained sequences is count data, probability of error is small while a large count of events is being observed. The statistics requires the rate of observed error, which was calculated. The error ratio (false results in index) was almost comparable to reported MinION native barcode crosstalk rate of 0.056%^25^. For the threshold calculation, the error ratio was multiplied by ten as a safety margin. This was an arbitrary value; however, it will reduce false positives and can be adjusted along with further data acquisition. Collectively, this threshold can differentiate potential cross-talk reads from neighbouring samples as demonstrated in the clinical samples.

In the validation study using clinical samples from Vietnam, the sensitivity and specificity of the Nanopore tests (Flongle and MinION combined) compared to the DENV RT-qPCR, were 66.7% and 95.4%, respectively. At present, the sensitivity, and specificity of broad range flavivirus NAT is limited. Several pan-flavivirus assays have been developed, but have lacked clinical validation^7, 26,27^. The DENV 1–4 RT-qPCR itself was reported to have sensitivity of 97.92%, using sequencing as a comparison^28^. When the Flongle/MinION test was compared to DENV 1–4 RT-qPCR, the sensitivity was rather satisfactory at 66.7%; however, samples with low Ct values were also positive and in contrast, those with high Ct values were negative in the Flongle/MinION test. Additionally, in this study the RT-qPCR test could only detect six out of ten positive samples while MinION detected eight out of ten positive samples (Table 2). Indeed, when RT-qPCR was compared to Nanopore detection system, the sensitivity and specificity were 57.14% and 96.88%, respectively. It is important to mention that RT-qPCR is not a gold standard test, and cross comparison showed a similar sensitivity (66.7% and 57.14%). Therefore, it can be concluded that Nanopore test has comparable performance to the adopted RT-qPCR for Dengue as provided by the CDC.

In contrast, the observed sensitivity was low when compared to the NS1 antigen test (Table 3). A previous study reported that the sensitivity of NS1 ELISA test is around 83.6%^29^. This can be mainly attributed to the outstanding performance of the NS1 test, different methods of detection, and last but not least to the detectable window of antigen and viral RNA. The NS1 antigen can be detected from first to the ninth day (sixth day in secondary infection) after fever onset and can remain in blood two or three days longer after viremia, providing a longer window period for detection^30,31^. Our method was able to detect one positive sample from the NS1 negative group (Table 2). This discrepancy might be accounted for a lesser sensitivity of NS1 detection, particularly in secondary infection and in DENV4 infection as previously reported^32^.

Another advantage of the pan-flavivirus-Flongle/MinION system is the broad comprehensiveness for the genus flavivirus. This was demonstrated by the detection of nine different flaviviruses using plasmid templates (Fig. 1a, Supplementary Tables S4 and S5). In addition, we successfully detected DENV1, 2, and 4 from clinical samples in Vietnam. Besides, SLEV, YFV, WNV, DENV, and ZIKV were detected from clinical samples in Brazil (Supplementary Table S10). This was a pilot study conducted before the study in Vietnam and a primer set with shorter index sequence (14-nt) was used. In case of metagenomic NGS, at least 50 reads or more than 10 reads per million ratio of sample to negative control have been used for threshold^33–35^. In amplicon sequencing, singletons are filtered in general. Whichever criteria was applied, SLEV, YFV, WNV, DENV, and ZIKV were specifically detected in the samples. This supports the feasibility of broad detection of flavivirus from clinical samples using the pan-flavivirus-Flongle/MinION system.

Infectious disease outbreaks in recent years have highlighted the importance of molecular detection, as well as sequencing being an inseparable part in control efforts. In this study, a combination of Nanopore sequencing and pan-flavivirus RT-PCR has shown its merit for broad detection of flaviviruses. The current molecular approaches for flavivirus detection lack broad specificity, while the serology tests suffer from similar issues as well as cross reactivity between flavivirus species. The method described in this paper, however, has been shown to overcome those limitations. To increase the sample throughput from the sequencing test, a dual index system was developed with a tailored bioinformatics pipeline to minimize crosstalk between samples. The dual-index system can greatly improve the sample throughput, allowing up to 144 samples to be multiplexed and run in one MinION flowcell. The sequencing test was also compared to a commercially available NAT kit, in which the Nanopore test detected more positive samples. Other merits of using Nanopore is that this workflow can be applied in resource limited settings, provides access to real time sequencing data for rapid identification, and a much reduced cost for sequencing. One downside of the Nanopore test, particularly the multiplexing system, is that the index sequence is hampering the PCR reaction through primer dimer formation. Nevertheless, the new R10.3 flowcell from Nanopore with improved raw sequencing accuracy should allow a shorter index to be used, and subsequently improve the detection sensitivity. Taken altogether, the pan-flavivirus-Nanopore detection system can be expected to provide a considerable contribution to the detection and surveillance of flaviviruses.

## Materials and methods

### Primer modifications

Filled/truncated right end edit (FREE) software were used to generate the oligonucleotide set^11^. A set of 14-mer-long and 12-mer-long oligonucleotides were generated then concatenated using the same tool. Sequences with homopolymer (more than 2 bases) were excluded. The concatenated sequences were then aligned to each other with LAST^12^ and index with alignments score more than 40 were excluded. Different index sequences (14-mer-long) were used in the experiments for Brazilian samples.

### Primer validations with in vitro-spiked sample

The modified primers were validated using serially diluted fetal bovine serum (FBS) spiked with viral particles of DENV1, DENV2, and YFV17D to a final concentration ranging from 10 to 10^5^ PFU/mL. Viral RNA was extracted from samples using QIAamp viral RNA mini kit (QIAGEN), according to the manufacturer’s instruction. One-step RT-PCR was carried out using PrimeScript One Step RT-PCR Kit Ver.2 (Takara) according to the manufacturer’s instruction, in a total reaction of 15 µL containing 2 µL template RNA, 250 nM of each sense primers, and 500 nM anti-sense primer. Temperature conditions for the RT-PCR was as follows: 50 °C for 30 min (cDNA synthesis), 94 °C for 30 s, followed by 43 cycles of 94 °C, 53 °C, 72 °C, 30 s each, and lastly 72 °C for 5 min. The amplicons (1 µL) were then visualized on 1.5% agarose gels alongside Gene Ladder100, a 100 bp DNA marker (Nippongene).

### Nanopore sequencing

Amplicons were purified with 1 × AMpureXP beads (Beckman Coulter). The DNA concentrations of each sample were measured with Qubit fluorometer (Invitrogen). End-repair and dA-tailing was performed with the II end-prep reaction buffer and enzyme mix (New England Biolabs), then purified with 1 × AMPureXP beads. Library construction was carried out using library kit SQK-109 (Oxford Nanopore). In addition, to check whether there is cross contamination between the index, each indexed sample was also barcoded with the Oxford native barcode. Barcode kit NBD103 and NBD114 (Oxford Nanopore) were used in these experiments. Ligation and tethering of the native barcode were carried out using 25 µL Blunt/TA ligase master mix and 2.5 µL native barcode (New England Biolabs) and then purified with 1 × AMPureXP beads. Equimolar amounts of barcoded samples were pooled and eluted with water in a total volume of 60 µL, prior to adapter ligation. For adapter ligation and tethering, the reaction was carried out with 20 µL Barcode Adapter Mix, 20 µL NEB Next Quick Ligation Reaction Buffer (5X), and 10 µL Quick T4 DNA Ligase. The barcoded and adapter-ligated amplicons was then purified with AMPureXP beads, washed with Short Fragment Buffer, and finally eluted in the elution buffer. The Flowcell (version 9.4) was primed with 1 mL mix of 30 µL Flush Tether and 1 tube of Flush Buffer. Twelve µL of cDNA library, 37.5 µL sequencing buffer and 22.5 µL loading beads were mixed and loaded into the flowcell.

For sequencing with Flongle, barcoded samples were pooled in a final of 32.5 µL. Adapter ligation was carried out using 2.5 µL Adapter Mix II, 10 µL NEB Next Quick Ligation Reaction Buffer (5X), and 5 µL Quick T4 DNA Ligase, which was then purified with 1 × AMPureXP beads. The flowcell (version 9.4) was primed with 100 µL of 3 µL Flush Tether and 117 µL of Flush Buffer mix. Five µL of cDNA library, 15 µL sequencing buffer and 10 µL loading beads were mixed and loaded into the flowcell.

### Bioinformatic analysis

Three different sequencing data (one from MinION and two from Flongle) were analysed with each pipeline where different parameters were applied according to the tools. Raw FAST5 files from the nanopore instrument were basecalled with Guppy v 3.0 (Oxford Nanopore Technologies). Only reads with q-score more than seven were proceeded for downstream analysis. Debarcoding was carried out using Guppy v 3.0 were with stricter parameter applied (barcode score 90) to avoid cross-assigning reads as previously reported^25^. Different demultiplexing pipelines were tested with the reads, including FREEBarcodes^11^, LAST^12^, and Minibar^13^.

For the LAST-based pipeline, a database containing the index sequences (including the primer sequence) was generated using the option -uNEAR -R01. The debarcoded reads were then aligned with the database using following options: -Q1 -q2 -r2 -a1 -b1 -e 20. From the analysis, the reads were selected based on the score of the alignment. A script was written to select the read with certain score threshold (alignment score of 70). The reads were then demultiplexed to a separated fastq file based on the matched forward and reverse index. When using Minibar, the following options were applied: -F -e 4 -l 100 -P "".

The reads were filtered by length; only reads with 250–500 bp long were included in the next analysis. The deindexed reads were then converted into FASTA files for alignment searches using BLAST (basic local alignment search tool)^36^. A local virus database which was constructed for READSCAN was used for the BLAST search^37^. Reads were then filtered based on percentage identity (above 80%) and alignment length (250–290). The results were classified into true or false. The reads were classified as true if the read were classified into the correct pair of barcode, matched index, and virus (Supplementary Fig. S1b). As for false reads, the reads were classified into false in barcode if the read has match index pair, and virus, but were binned in an unmatched barcode. Reads that matched the barcode assigned and the virus, with mismatched index combination were assigned as false in index. When the reads were assigned into a barcode with matched index combination but contained sequences other than the viruses contained in the sample, these were classified as false in sequencing.

### Threshold calculation

Results with very limited read numbers (one or two reads) are automatically omitted from the result. When read counts are more than two, with the assumption that false in index rate would be constant, the reliability of the detection, i.e. threshold for positive or negative results, was calculated based on the error rate. We also assume that error in index happen only in one of the two indices; thus cross-talk sequences come from neighbouring samples with identical forward or reverse indexes. Estimated reads cross-talk were calculated based on the error rate with a safety margin of 10 times to minimalize false positive result. The expected amount of cross-talk was plotted to a Poisson distribution. In the event (µ) where the upper cumulative Poisson probability (P(x ≥ µ)) was less than 0.01 was determined as the threshold. The calculation was based only on reads that have BLAST hit and counted at the result. If the number of reads from a certain virus passed the cross-talk threshold, the sample will be considered positive.

### System validation with plasmids

Viral RNA (DENV1, DENV2, DENV3, DENV4, JEV, YFV, WNV, ZIKV, TBEV) was subjected to RT-PCR as described above, except the primer contain no index sequences.

The amplicons were then cloned into pGEM-T vectors (Promega) and transformed into *Escherichia coli* DH5a. Overnight grown colonies containing plasmid with insertions were then allowed to proceed to expansion for 12 h. Plasmids were then purified using Wizard Plus SV Minipreps (Promega) according to the manufacturer’s instruction. Nucleic acid quantification was performed with a Qubit fluorometer (Invitrogen). The purified plasmids were then diluted to achieve plasmid concentration ranging from 10^0^ to 10^6^ plasmids/µl. This serially diluted plasmid was then subjected to PCR using identical polymerase used in the one-step RT-PCR (TaKaRa ex Taq Hot Start (Takara)) according to the manufacturer’s instructions. The PCR was carried in a total reaction volume of 15 µL containing 1 µL template, 250 nM each of indexed sense primers, 500 nM anti-sense primer. Cycling program was as follows: 94 °C for 30 s, followed by 43 cycles of 94 °C, 53 °C, 72 °C, 30 s each, and lastly 72 °C for 5 min. Amplicons (1 µL) were then visualized in 1.5% agarose gel, along with Gene Ladder100 (Nippongene). Amplicons obtained from reaction with the following concentration of template: 10^3^ cp/reaction for DENV1, DENV2, DENV3, JEV, YFV, TBEV, ZIKV, and WNV and 10^2^ cp/reaction for DENV4, which resulted in visible band at the gel (by naked eye) were sequenced with Flongle. Furthermore, amplicons obtained from 10- or 100-times dilution from the concentration above (representing samples with no visible bands) were also sequenced with MinION which has more pores; therefore, permitting deep sequencing.

### Clinical samples

Clinical samples were provided by the National Institute of Hygiene and Epidemiology (NIHE) Vietnam. A total of 114 serum samples were examined with this system. The samples were stored at − 80 °C prior to the experiment. All samples were subjected to NS1 antigen test (Inbios). The NS1 positive sample were also subjected to DENV-1-4 RT-qPCR Multiplex Assay as described previously^28^. The samples from Vietnam were subjected to one step RT-PCR with indexed primer as mentioned above, with each sample assigned to different index combination (total 114 combinations). All samples (including RT-PCR negative samples) were then pooled and sequenced with the Flongle flowcell. Subsequently, a second round of sequencing with MinION was carried out excluding Flongle-positive samples. Second set of clinical samples were also obtained from Flavivirus Laboratory, the Oswaldo Cruz Foundation, Rio de Janeiro, Brazil. A total of 24 serum samples were examined with this system. These samples were examined with a flavivirus RT-qPCR as described previously^38^. Positive samples were then subjected to virus-specific protocols as previously described^28,39–42^. Different sets of index sequences were used to analyse these samples (Supplementary Table S2). The raw sequencing data of this study has been submitted to National Center for Biotechnology Information (NCBI) database under the BioProject PRJNA745522.

### Ethical statement

For samples from Vietnam, ethical permission was obtained both from NIHE (VSDT18/2018) and Hokkaido University (Jinjyu1-3). For samples from Brazil, ethical permissions were obtained from Oswaldo Cruz Foundation (no. 2.998.362) and Hokkaido University (Jinjyu30-4).

## Supplementary Information

Supplementary Information.
