# Supplementary material for: A targeted approach with nanopore sequencing for the universal detection and identification of flaviviruses
Source: Sci Rep. 2021 Sep 24;11:19031. doi: 10.1038/s41598-021-98013-9 (PMC8463598; doi:10.1038/s41598-021-98013-9)
Supplement: Supplementary file 1 — Supplementary Information. [file 41598_2021_98013_MOESM1_ESM.pdf]

Supplementary material

A Targeted Approach with Nanopore Sequencing for the Universal Detection and Identification of Flaviviruses

Patrick Reteng[1], Linh Nguyen Thuy[2], Tran Thi Minh Tam[2], Maria Angélica Monteiro de Mello Mares-Guia[3], Maria Celeste Torres[3], Ana Maria Bispo de Filippis[3], Yasuko Orba[4], Shintaro Kobayashi[5], Kyoko Hayashida[1,4], Hirofumi Sawa[4], William W. Hall[5,6,7], Lan Anh Nguyen Thi[2], Junya Yamagishi[1,4,*]

[1] Division of Collaboration and Education, International Institute for Zoonosis Control, Hokkaido University, Sapporo, Japan

[2] Center for Bio-Medical Research, National Institute of Hygiene and Epidemiology, Hanoi, Vietnam.

[3] Flavivirus Laboratory, Oswaldo Cruz Institute, Fiocruz, Rio de Janeiro, Brazil

[4] Division of Molecular Pathobiology, International Institute for Zoonosis Control, Hokkaido University, Sapporo, Japan

[5] Laboratory of Public Health, Faculty of Veterinary Medicine, Hokkaido University, Sapporo, Japan

[6] International Collaboration Unit, International Institute for Zoonosis Control, Hokkaido University, Sapporo, Japan

[7] Global Virus Network, Baltimore, United States of America

[8] National Virus Reference Laboratory, University College Dublin, Dublin, Ireland

[9] Ireland Vietnam Blood-Borne Virus Initiative (IVVI), Dublin, Ireland.

[*]junya@czc.hokudai.ac.jp

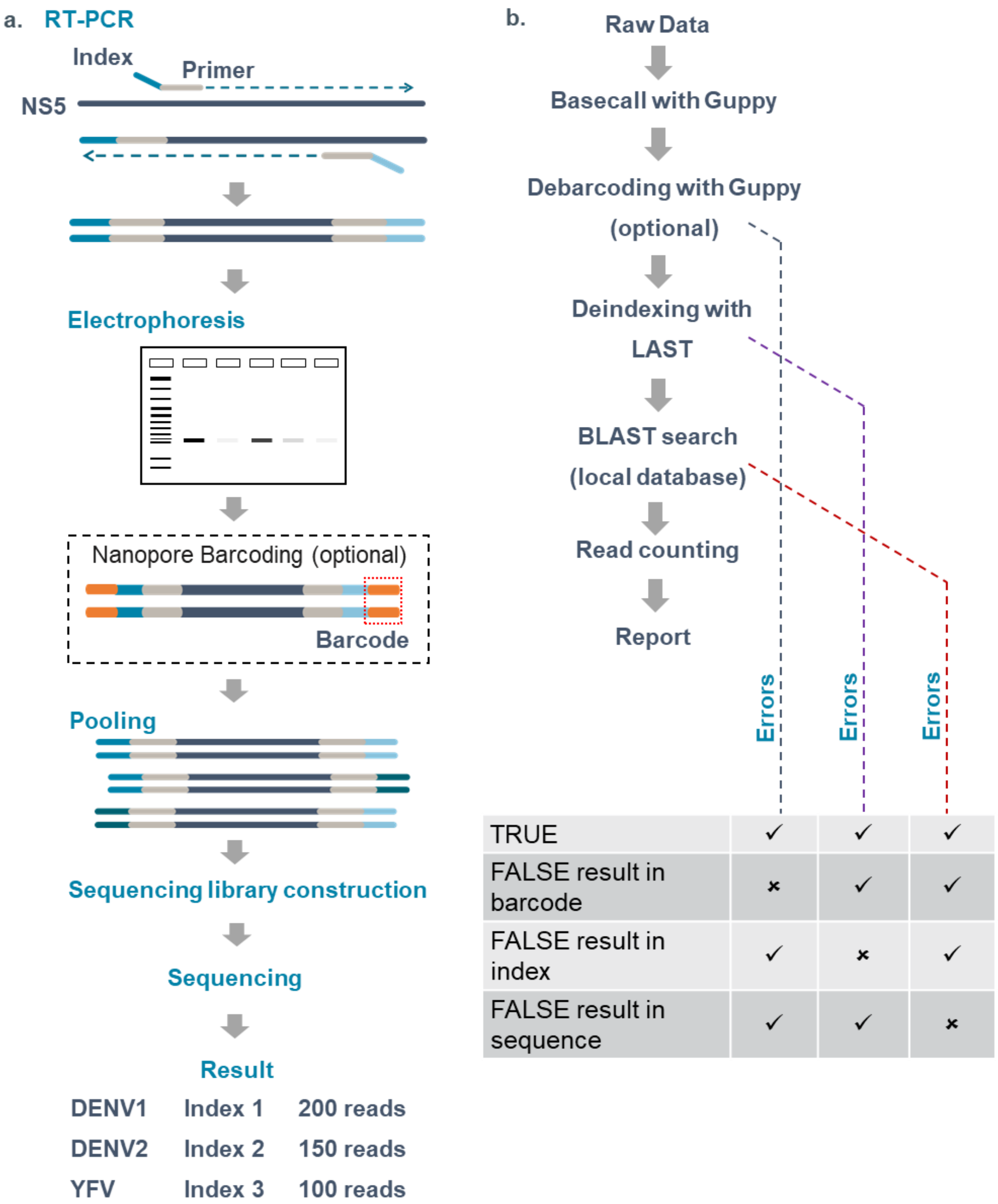

Supplementary Figure S1. Workflow for sequencing experiment. (A) Pan-flaviviurs PCR is conducted using modified primers with index. Amplicons are pooled then subjected to sequencing using Nanopore, respectively. (B) Bioinformatic workflow to analyze the sequences. Errors were classified based on the properties assigned to each read (barcode, index, and virus sequence).

a. Sample 02, DENV2 reads

	i13	i14	i15	i16	i17	i18	i19	i20	i21	i22	i23	i24
i01												
i02		1,055										
i03												
i04												
i05					1							
i06												
i07		1										
i08												
i09												
i10												
i11												
i12												

$$\lambda(\text{i02}, \text{i14}) = (1056 \times 0.02\%) \times 10 = 2.11$$

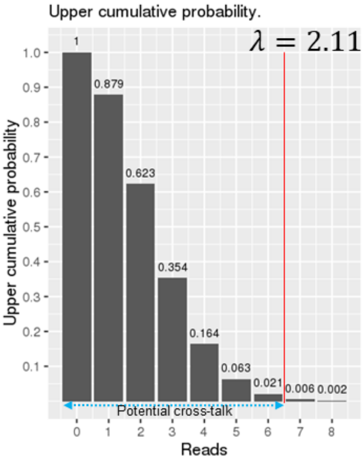

b. DENV1 reads obtained from Vietnam sample (Flongle sequencing)

	i13	i14	i15	i16	i17	i18	i19	i20	i21	i22	i23	i24
i01							3	3				
i02								2				
i03								1				
i04								1				
i05							1	1				
i06				1	1		179		3		43	1
i07								1				
i08											1	
i09							1	2				
i10							1					
i11	1			272		6	1	4	2			
i12			1	1			50	370	2	1		

$$\lambda(i01, i19) = (239 \times 0.02\%) \times 10 = 0.48$$

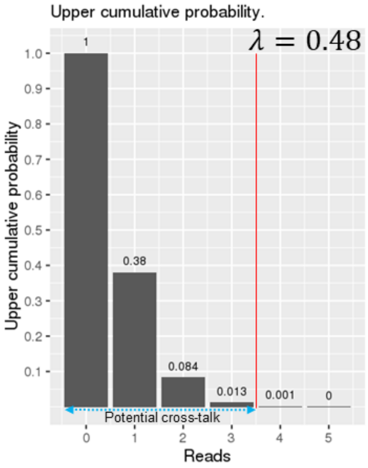

Supplementary Figure S2. Calculation of threshold for potential cross-talk. Threshold was calculated based on assumption that cross-talk reads originate from neighbouring samples with identical forward or reverse index. Error rate with a safety margin of ten times was used to estimate the λ value. The value was then plotted to Poisson cumulative distribution. In the event (μ) where the upper cumulative Poisson probability ($P(x \geq \mu)$) was less than 0.01 was determined as the threshold. (A) Calculation of cross-talk threshold for i07-i14, showed that seven reads are needed for the result to be classified as positive. (B) Similar calculation was performed for clinical sample. The obtained 3 reads with i01-i19 index pair were classified as negative.

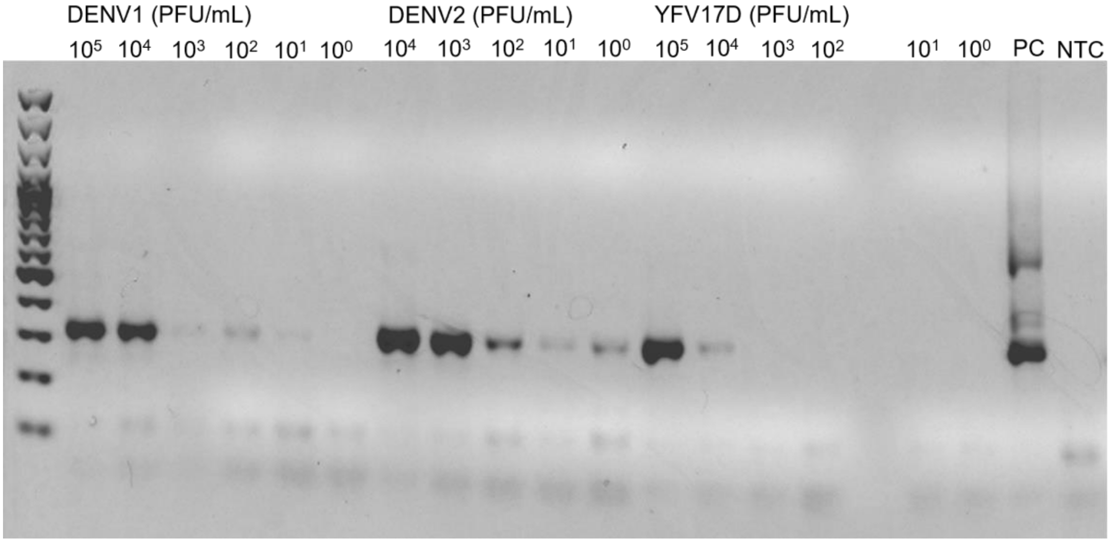

Supplementary Figure S3. Gel electrophoresis image obtained from serial dilution experiment. Fetal bovine serum was spiked with viral particles then diluted accordingly to 1 PFU/mL. Viral RNA were extracted, then subjected to pan-flavivirus RT-PCR with indexed primer.

Fa

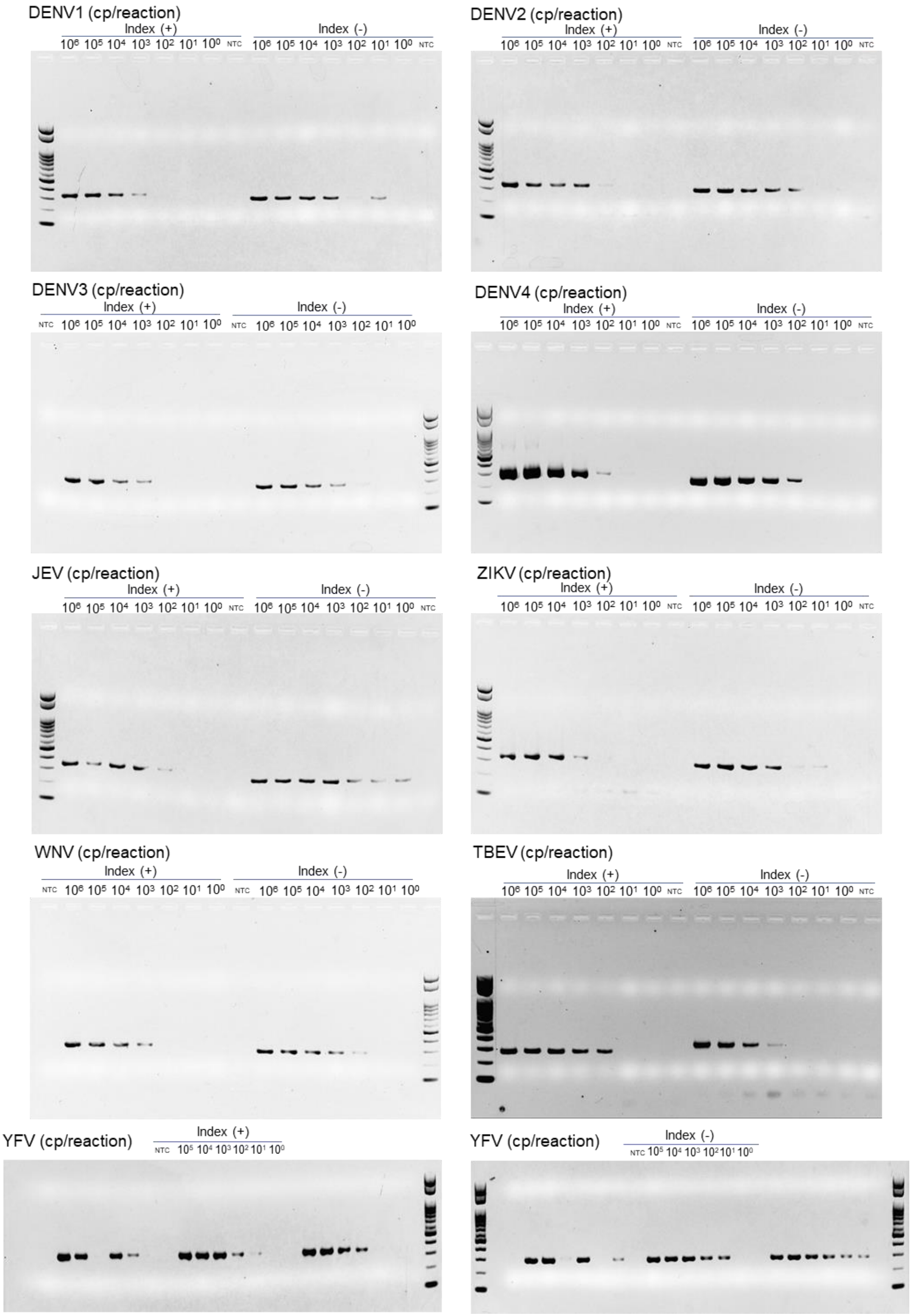

Supplementary Figure S4. Gel electrophoresis image obtained from serial dilution experiment using plasmid containing viral sequences as template

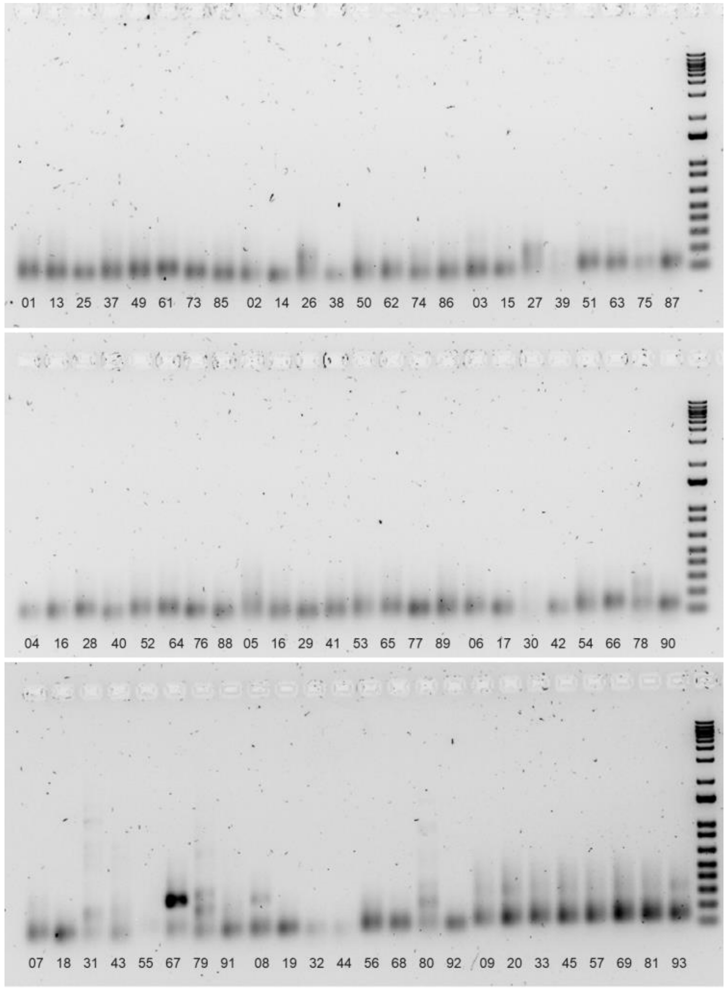

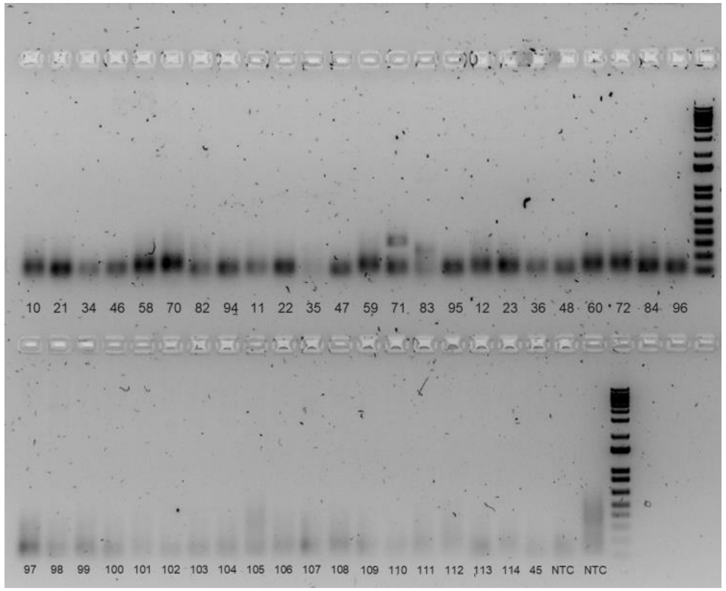

	i13	i14	i15	i16	i17	i18	i19	i20	i21	i22	i23	i24
i01	1	2	3	4	5	6	7	8	9	10	11	12
i02	13	14	15	16	17	18	19	20	21	22	23	24
i03	25	26	27	28	29	30	31	32	33	34	35	36
i04	37	38	39	40	41	42	43	44	45	46	47	48
i05	49	50	51	52	53	54	55	56	57	58	59	60
i06	61	62	63	64	65	66	67	68	69	70	71	72
i07	73	74	75	76	77	78	79	80	81	82	83	84
i08	85	86	87	88	89	90	91	92	93	94	95	96
i09	97	98	99	100	101	102	103	104	105	106	107	108
i10	109	110	111	112	113	114	45					

Positive for NS1 **but negative** for DENV1-4 qPCR.
Positive for **both** NS1 and DENV1-4 qPCR.
Negative for NS1 and were **not** subjected to DENV1-4 qPCR.
Sample set from experiment not included in this study.
Unused index combination.

Supplementary Figure S5. Visualization of the panflavivirus RT-PCR result of the 114 samples collected in Vietnam. Sample id number can be seen at the bottom of the picture and the corresponding forward index (row) and reverse index (column) can be seen in the matrix.

Supplementary Table S1a. List of primer sequences used in this study.

Name	Sequence	Index length
Flavi_all_S_i01	ATTCTCTGGATCTCAAGCGGTCATTATACAACATGATGGGGAARAGAGARAA	26
Flavi_all_S_i02	AATGGTTCACACTCAAGGATACTCTCTACAACATGATGGGGAARAGAGARAA	26
Flavi_all_S_i03	AACCGAGAATTAGGAAGTCACACGCCTACAACATGATGGGGAARAGAGARAA	26
Flavi_all_S_i04	GTTCAGACAGTATGACAAGAGATTCCTACAACATGATGGGGAARAGAGARAA	26
Flavi_all_S_i05	CACTTATATCACGGACCTCCTGCGAATACAACATGATGGGGAARAGAGARAA	26
Flavi_all_S_i06	ACGGTCTATTGTTGAGTATCTGGTGATACAACATGATGGGGAARAGAGARAA	26
Flavi_all_S_i07	AACAACAACAACCGATGCACCTCTGTTACAACATGATGGGGAARAGAGARAA	26
Flavi_all_S_i08	TGCCATCTTGCGGAATTCATACCAGCTACAACATGATGGGGAARAGAGARAA	26
Flavi_all_S_i09	ACCATTGACCATAGCACTTCCGAGCTTACAACATGATGGGGAARAGAGARAA	26
Flavi_all_S_i10	TGGTGGTCGCTGAACCGGTGAGTTAATACAACATGATGGGGAARAGAGARAA	26
Flavi_all_S_i11	CCTAACAATTCTGCCGTACCGGACAATACAACATGATGGGGAARAGAGARAA	26
Flavi_all_S_i12	CCGAAGCTCCACTACTAACCATGAAGTACAACATGATGGGGAARAGAGARAA	26
DEN4_F_i01	ATTCTCTGGATCTCAAGCGGTCATTATACAACATGATGGGRAAACGTGAGAA	26
DEN4_F_i02	AATGGTTCACACTCAAGGATACTCTCTACAACATGATGGGRAAACGTGAGAA	26
DEN4_F_i03	AACCGAGAATTAGGAAGTCACACGCCTACAACATGATGGGRAAACGTGAGAA	26
DEN4_F_i04	GTTCAGACAGTATGACAAGAGATTCCTACAACATGATGGGRAAACGTGAGAA	26
DEN4_F_i05	CACTTATATCACGGACCTCCTGCGAATACAACATGATGGGRAAACGTGAGAA	26
DEN4_F_i06	ACGGTCTATTGTTGAGTATCTGGTGATACAACATGATGGGRAAACGTGAGAA	26
DEN4_F_i07	AACAACAACAACCGATGCACCTCTGTTACAACATGATGGGRAAACGTGAGAA	26
DEN4_F_i08	TGCCATCTTGCGGAATTCATACCAGCTACAACATGATGGGRAAACGTGAGAA	26
DEN4_F_i09	ACCATTGACCATAGCACTTCCGAGCTTACAACATGATGGGRAAACGTGAGAA	26
DEN4_F_i10	TGGTGGTCGCTGAACCGGTGAGTTAATACAACATGATGGGRAAACGTGAGAA	26
DEN4_F_i11	CCTAACAATTCTGCCGTACCGGACAATACAACATGATGGGRAAACGTGAGAA	26
DEN4_F_i12	CCGAAGCTCCACTACTAACCATGAAGTACAACATGATGGGRAAACGTGAGAA	26
Flavi_all_AS_13	CGTCTTCCTCCATACTCTTAGCAGTAGTGTCCCAGCCNGCKGTGTCATCWGC	26
Flavi_all_AS_14	CGTTGTGAGGTGGAGATTATGGTACGGTGTCCCAGCCNGCKGTGTCATCWGC	26
Flavi_all_AS_15	TTATGCCAATCGGAGCCTGACGCTTAGTGTCCCAGCCNGCKGTGTCATCWGC	26
Flavi_all_AS_16	AACACCGCTCTTCTGCTGCAATATACGTGTCCCAGCCNGCKGTGTCATCWGC	26
Flavi_all_AS_17	CTCCGCATTAACTGGGTGGTGACAAGGTGTCCCAGCCNGCKGTGTCATCWGC	26
Flavi_all_AS_18	ACGCCACCTAAGATGTATTCCAGATGGTGTCCCAGCCNGCKGTGTCATCWGC	26
Flavi_all_AS_19	CGTAGGAACCAAGAGTCGCCACAACTGTGTCCCAGCCNGCKGTGTCATCWGC	26
Flavi_all_AS_20	AGTAAGCCTGTGCCTAGTGACGATGGGTGTCCCAGCCNGCKGTGTCATCWGC	26
Flavi_all_AS_21	CAGGAGTGTCTTAATATCATTCGCTCGTGTCCCAGCCNGCKGTGTCATCWGC	26
Flavi_all_AS_22	AAGGTAAGAGAAGGTCGAAGCGCGTTGTGTCCCAGCCNGCKGTGTCATCWGC	26
Flavi_all_AS_23	AAGTTGCGGTTATGTGAGAGCCTATTGTGTCCCAGCCNGCKGTGTCATCWGC	26
Flavi_all_AS_24	AAGAATGTATCGCCTTATGTGCTGCGGTGTCCCAGCCNGCKGTGTCATCWGC	26

Supplementary Table S1b. List of primer sequences used for experiment using clinical samples from Brazil.

Name	Sequence	Index length
Flavi_all_S_i01	CTATACAGCATGAGTACAACATGATGGGGAARAGAGARAA	14
Flavi_all_S_i02	AGAGTCTAGCTAGCTACAACATGATGGGGAARAGAGARAA	14
Flavi_all_S_i03	TGCGACACATGTGATACAACATGATGGGGAARAGAGARAA	14
Flavi_all_S_i04	GACTATGCAGTGCATACAACATGATGGGGAARAGAGARAA	14
Flavi_all_S_i05	ACGCGTGCATCTACTACAACATGATGGGGAARAGAGARAA	14
Flavi_all_S_i06	TCGAGTAGTCTCAGTACAACATGATGGGGAARAGAGARAA	14
Flavi_all_S_i07	GTATCATGTCAGCATACAACATGATGGGGAARAGAGARAA	14
Flavi_all_S_i08	AGCTAGTAGCTACTTACAACATGATGGGGAARAGAGARAA	14
Flavi_all_S_i09	CGAGACGATACTCTTACAACATGATGGGGAARAGAGARAA	14
Flavi_all_S_i10	TAGATGCTCGCGAGTACAACATGATGGGGAARAGAGARAA	14
Flavi_all_S_i11	GCTACGCTGAGTAGTACAACATGATGGGGAARAGAGARAA	14
Flavi_all_S_i12	TCTCAGCGCAGTGATACAACATGATGGGGAARAGAGARAA	14
DEN4_F_i01	CTATACAGCATGAGTACAACATGATGGGRAAACGTGAGAA	14
DEN4_F_i02	AGAGTCTAGCTAGCTACAACATGATGGGRAAACGTGAGAA	14
DEN4_F_i03	TGCGACACATGTGATACAACATGATGGGRAAACGTGAGAA	14
DEN4_F_i04	GACTATGCAGTGCATACAACATGATGGGRAAACGTGAGAA	14
DEN4_F_i05	ACGCGTGCATCTACTACAACATGATGGGRAAACGTGAGAA	14
DEN4_F_i06	TCGAGTAGTCTCAGTACAACATGATGGGRAAACGTGAGAA	14
DEN4_F_i07	GTATCATGTCAGCATACAACATGATGGGRAAACGTGAGAA	14
DEN4_F_i08	AGCTAGTAGCTACTTACAACATGATGGGRAAACGTGAGAA	14
DEN4_F_i09	CGAGACGATACTCTTACAACATGATGGGRAAACGTGAGAA	14
DEN4_F_i10	TAGATGCTCGCGAGTACAACATGATGGGRAAACGTGAGAA	14
DEN4_F_i11	GCTACGCTGAGTAGTACAACATGATGGGRAAACGTGAGAA	14
DEN4_F_i12	TCTCAGCGCAGTGATACAACATGATGGGRAAACGTGAGAA	14
Flavi_all_AS_i13	TAGCTCGACTGCGAGTGTCCCAGCCNGCKGTGTCATCWGC	14
Flavi_all_AS_i14	AGCTGCTGATCACTGTGTCCCAGCCNGCKGTGTCATCWGC	14
Flavi_all_AS_i15	TGACAGTCAGTCGTGTGTCCCAGCCNGCKGTGTCATCWGC	14
Flavi_all_AS_i16	GCATGTGTATACACGTGTCCCAGCCNGCKGTGTCATCWGC	14
Flavi_all_AS_i17	CTCGCATCGATGCAGTGTCCCAGCCNGCKGTGTCATCWGC	14
Flavi_all_AS_i18	TATGAGATCTGCTCGTGTCCCAGCCNGCKGTGTCATCWGC	14
Flavi_all_AS_i19	GACGTCATAGTGCAGTGTCCCAGCCNGCKGTGTCATCWGC	14
Flavi_all_AS_i20	TCGATCGCGCATAGGTGTCCCAGCCNGCKGTGTCATCWGC	14
Flavi_all_AS_i21	TAGTACTGTGACACGTGTCCCAGCCNGCKGTGTCATCWGC	14
Flavi_all_AS_i22	CATGCACTGATCGTGTGTCCCAGCCNGCKGTGTCATCWGC	14
Flavi_all_AS_i23	ACTCTGTCACGTGAGTGTCCCAGCCNGCKGTGTCATCWGC	14
Flavi_all_AS_i24	CTACGTGCGCTAGAGTGTCCCAGCCNGCKGTGTCATCWGC	14

Supplementary Table S2. The mean recovery reads, true result, and false result from the bioinformatic analysis using three different tools and various parameters. [a]Percentage of input reads to reads with decoded index (number of deindexed reads/number of input reads). [b]True results are reads with correct barcode, index, and virus combination. [c] False results in barcode are deindexed reads with matched index and virus combination but were binned into wrong barcode. [d]False results in index are deindexed reads with matched barcode and virus combination but were binned into index pair not included in the sample. [e]False results in sequencing represent reads with matched index and barcode but hit other sequences beside the virus used in the study. Percentage was calculated by dividing the number of reads with total of true and false reads.

Tools	Parameter	Recovery Rate[a] (%)				TRUE[b] (%)				False result in barcode[c] (%)				False result in index[d] (%)				False result in sequencing[e] (%)			
		MinION	Flongle 1	Flongle 2	Average	MinION	Flongle 1	Flongle 2	Average	MinION	Flongle 1	Flongle 2	Average	MinION	Flongle 1	Flongle 2	Average	MinION	Flongle 1	Flongle 2	Average
LAST	Score - 60	41.78	49.05	53.29	48.04	98.89	99.27	99.19	99.12	0.63	0.17	0.45	0.42	0.44	0.50	0.22	0.39	0.04	0.06	0.14	0.08
	Score - 65	31.97	38.89	41.63	37.50	99.17	99.60	99.37	99.38	0.67	0.25	0.44	0.45	0.12	0.08	0.05	0.08	0.05	0.07	0.14	0.09
	Score - 70	22.50	28.62	29.46	26.86	99.32	99.70	99.33	99.45	0.60	0.20	0.49	0.43	0.01	0.02	0.03	0.02	0.07	0.07	0.14	0.09
	Score - 75	14.05	18.92	18.56	17.18	99.29	99.70	99.28	99.42	0.63	0.24	0.55	0.47	0.00	0.00	0.01	0.00	0.08	0.07	0.16	0.10
	Score - 80	7.55	10.88	9.56	9.33	99.22	99.59	99.28	99.36	0.69	0.33	0.55	0.52	0.00	0.00	0.01	0.00	0.09	0.08	0.16	0.11
	Score - 85	2.86	4.52	3.48	3.62	98.54	99.65	99.18	99.12	1.19	0.26	0.62	0.69	0.00	0.00	0.00	0.00	0.28	0.09	0.20	0.19
	Score - 90	0.37	0.76	0.54	0.56	97.22	99.40	99.67	98.76	2.78	0.00	0.33	1.04	0.00	0.00	0.00	0.00	0.00	0.60	0.00	0.20
MINIBAR	Edit distance 6	28.53	37.76	49.47	38.59	95.77	95.06	98.15	96.33	0.58	0.17	0.47	0.41	3.57	4.50	1.24	3.10	0.08	0.27	0.14	0.16
	Edit distance 5	24.36	33.03	41.45	32.95	98.69	97.15	99.28	98.37	0.55	0.17	0.45	0.39	0.72	2.61	0.15	1.16	0.04	0.07	0.13	0.08
	Edit distance 4	19.53	27.15	32.79	26.49	98.67	97.14	99.27	98.36	0.57	0.18	0.46	0.40	0.72	2.64	0.16	1.17	0.04	0.04	0.11	0.06
	Edit distance 3	14.09	20.90	23.51	19.50	98.51	97.01	99.18	98.23	0.55	0.13	0.47	0.38	0.88	2.82	0.21	1.30	0.06	0.05	0.14	0.08
	Edit distance 2	8.81	13.45	14.27	12.18	98.80	97.37	99.10	98.42	0.51	0.13	0.55	0.40	0.61	2.46	0.27	1.11	0.08	0.04	0.08	0.07
FREEBARCODES	Edit distance 7	10.78	1.09	1.23	4.37	98.96	91.67	99.07	96.57	0.58	0.00	0.00	0.19	0.37	8.33	0.62	3.11	0.08	0.00	0.31	0.13
	Edit distance 5	9.92	1.19	1.18	4.10	99.43	91.67	99.74	96.95	0.57	0.00	0.00	0.19	0.00	0.00	0.00	0.00	0.00	0.00	0.26	0.09
	Edit distance 3	5.15	0.64	0.59	2.13	99.23	91.20	99.31	96.58	0.77	0.00	0.00	0.26	0.00	0.00	0.00	0.00	0.00	0.46	0.69	0.38

Supplementary Table S3. Distribution of reads binned to index and barcode from sequencing of spiked samples with MinION (A) and Flongle (B and C). The optimized pipeline (LAST, score 70) was applied to analyze the data. The tables showing the details of the number of debarcoded reads, deindexed reads, true results, and false results. Deindexed reads might be binned to the correct or wrong index pair; both are listed as deindexed reads and will be subjected to BLAST search. The reads were then classify into true or false as mentioned above. DENV: Dengue Virus, YFV: Yellow Fever Virus

A.

		MinION											
					Deindexed Reads						False result in sequencing		
**Sample no**	**Spiked virus**	**Barcode**	**Index**	**Debarcoded reads**	**Correct Index**	**Other Index**	**No Index**	**TRUE**	**False result in barcode**	**False result in index**	**Other Flaviviruses**	**Other viruses**	**Other**
					**(%)**	**(%)**	**(%)**	**(%)**	**(%)**	**(%)**	**(%)**	**(%)**	**(%)**
**Sample 01**	DENV1	1	i01	2,960	684	0	2,276	607	0	0	0	0	0
			i13		(23.11)	(0)	(76.89)	(100)	(0)	(0)	(0)	(0)	(0)
**Sample 02**	DENV2	2	i02	5,062	1,454	2	3,606	1,055	1	1	2	0	1
			i14		(28.72)	(0.04)	(71.28)	(99.53)	(0.09)	(0.09)	(0.19)	(0)	(0.09)
**Sample 03**	YFV	3	i03	3,066	397	9	2,660	350	8	0	0	0	0
			i15		(12.95)	(0.29)	(87.05)	97.77	(2.23)	0	(0)	(0)	(0)
**Sample 04**	DENV1	4	i04	5,312	2068	4	3,240	1,784	3	0	0	0	0
			i16		(38.93)	(0.08)	(61.07)	(99.83)	(0.17)	(0)	(0)	(0)	(0)
**Sample 05**	DENV2	5	i05	2,541	271	0	2,270	203	0	0	0	0	0
			i17		(10.67)	(0)	(89.93)	(100)	(0)	(0)	(0)	(0)	(0)
**Sample 06**	YFV	6	i06	5,942	1988	5	3,949	1,754	5	0	0	0	0
			i18		(33.46)	(0.08)	(66.54)	(99.72)	(0.28)	(0)	(0)	(0)	(0)
**Sample 07**	DENV1	7	i07	1,583	306	2	1,275	266	1	0	0	0	0
			i19		(19.33)	(0.13)	(80.67)	(99.63)	(0.37)	(0)	(0)	(0)	(0)
	DENV2	8	i08	1796	498	14	1,284	353	12	0	0	0	0

**Sample 08**			i20		(27.73)	(0.78)	(72.27)	(96.71)	(3.29)	(0)	(0)	(0)	(0)
**Sample 09**	YFV	9	i09<br>i21	1978	506<br>(25.58)	0<br>(0)	1,472<br>(74.42)	457<br>(100)	0<br>(0)	0<br>(0)	0<br>(0)	0<br>(0)	0<br>(0)
**Sample 10**	DENV1	10	i10<br>i22	2382	541<br>(22.71)	4<br>(0.17)	1,837<br>(77.29)	492<br>(99.19)	4<br>(0.81)	0<br>0	0<br>(0)	0<br>(0)	0<br>(0)
**Sample 11**	DENV2	11	i11<br>i23	4183	533<br>(12.74)	0<br>(0)	3,650<br>(87.26)	410<br>(99.51)	0<br>(0)	0<br>(0)	2<br>(0.49)	0<br>(0)	0<br>(0)
**Sample 12**	YFV	12	i12<br>i24	3253	456<br>(18.42)	0<br>(0)	2,797<br>(85.98)	403<br>(100)	0<br>(0)	0<br>(0)	0<br>(0)	0<br>(0)	0<br>(0)
**Average**					808.5<br>(22.49)	3.33<br>(0.13)	2,526.33<br>(77.50)	677.83<br>(99.32)	2.83<br>(0.60)	0.08<br>(0.01)	0.33<br>(0.06)	0<br>(0)	0.08<br>(0.01)

B.

Sample no	Spiked virus	Barcode	Index	Debarcoded reads[a]	Flongle 1: Deindexed Reads[b]: Correct Index (%)	Other Index (%)	No Index (%)	TRUE (%)	False result in barcode (%)	False result in index (%)	False result in sequencing: Other Flaviviruses (%)	Other viruses (%)	Other (%)
**Sample 01**	DENV1	1	i01	1,669	447	0	1,222	408	0	0	0	0	0
			i24		(26.78)	(0)	(73.22)	(100)	(0)	(0)	(0)	(0)	(0)
**Sample 02**	DENV2	5	i02	1,254	182	1	1,072	151	1	0	0	0	0
			i23		(26.92)	(0.15)	(73.08)	(99.34)	(0.66)	(0)	(0)	(0)	(0)
**Sample 03**	YFV	9	i03	964	362	1	602	329	1	0	0	0	0
			i22		(25.30)	(0.07)	(74.70)	(99.70)	(0.30)	(0)	(0)	(0)	(0)
**Sample 04**	DENV1	2	i04	1,237	312	2	925	276	2	0	0	0	0
			i19		(24.88)	(0.16)	(75.12)	(99.28)	(0.72)	(0)	(0)	(0)	(0)
**Sample 05**	DENV2	6	i05	676	483	3	193	400	1	1	1	0	0
			i20		(40.79)	(0.25)	(59.21)	(99.26)	(99.26)	(0.21)	(0.21)	(0)	(0)
**Sample 06**	YFV	10	i06	1,184	272	0	912	254	0	0	0	0	0
			i14		(27.39)	(0)	(72.61)	(100)	(0)	(0)	(0)	(0)	(0)
**Sample 07**	DENV1	3	i07	607	210	1	397	190	0	0	0	0	0
			i18		(21.78)	(0.10)	(78.22)	(100)	(0)	(0)	(0)	(0)	(0)
**Sample 08**	DENV2	7	i08	2,304	91	0	2,213	70	0	0	0	0	0
			i17		(14.99)	(0)	(64.89)	(100)	(0)	(0)	(0)	(0)	(0)
**Sample 09**	YFV	11	i09	1,431	371	0	1,060	338	0	0	0	0	0
			i13		(35.30)	(0)	(74.70)	(100)	(0)	(0)	(0)	(0)	(0)
**Sample 10**	DENV1	4	i10	993	459	0	534	401	0	0	0	0	0
			i16		(37.11)	(0)	(62.89)	(100)	(0)	(0)	(0)	(0)	(0)

**Sample 11**	DENV2	8	i10<br>i21	1,051	809<br>(35.11)	3<br>(0.13)	242<br>(64.70)	618<br>(98.88)	3<br>(0.48)	0<br>(0)	4<br>(0.64)	0<br>(0)	0<br>(0)
**Sample 12**	YFV	12	i12<br>i15	1,010	273<br>(27.03)	0<br>(0)	737<br>(72.97)	243<br>(100)	0<br>(0)	0<br>(0)	0<br>(0)	0<br>(0)	0<br>(0)
**Average**				1,198.83	355.92<br>(28.62)	0.92<br>(0.07)	841.5<br>(71.38)	306.5<br>(99.70)	0.75<br>(0.20)	0.08<br>(0.02)	0.42<br>(0.07)	0<br>(0)	0<br>(0)

C.

Sample no	Spiked virus	Barcode	Index	Debarcoded reads[a]	Flongle 2								
					Deindexed Reads[b]			TRUE	False result in barcode	False result in index	False result in sequencing		
					Correct Index	Other Index	No Index				Other Flaviviruses	Other viruses	Other
					(%)	(%)	(%)	(%)	(%)	(%)	(%)	(%)	(%)
**Sample 01**	DENV1	1	i01	6,632	2,291	2	4,339	1,872	2	0	0	0	0
			i14		(34.54)	(0.03)	(65.46)	(99.89)	(0.11)	(0)	(0)	(0)	(0)
**Sample 02**	DENV2	2	i02	5,243	1,711	6	3,526	1,187	4	1	0	0	0
			i15		(32.63)	(0.11)	(67.37)	(99.58)	(0.34)	(0.08)	(0)	(0)	(0)
**Sample 03**	YFV	3	i03	3,970	1,085	1	2,884	943	1	0	7	0	0
			i16		(27.33)	(0.03)	(72.67)	(99.16)	(0.11)	(0)	(0.74)	(0)	(0)
**Sample 04**	DENV1	4	i04	5,563	709	8	4,846	573	7	1	0	0	0
			i17		(12.74)	(0.14)	(87.26)	(98.62)	(1.20)	(0.17)	(0)	(0)	(0)
**Sample 05**	DENV2	5	i05	2,384	737	11	1,636	515	10	0	1	0	0
			i18		(30.91)	(0.46)	(69.09)	(97.91)	(1.90)	(0)	(0.19)	(0)	(0)
**Sample 06**	YFV	6	i06	687	155	0	532	134	0	0	0	0	0
			i19		(22.56)	(0)	(77.44)	(100)	(0.00)	(0)	(0)	(0)	(0)
**Sample 07**	DENV1	7	i07	4,163	1,666	1	2,496	1,388	1	0	0	0	0
			i20		(40.02)	(0.02)	(59.98)	(99.93)	(0.07)	(0)	(0)	(0)	(0)
**Sample 08**	DENV2	8	i08	8,422	3,254	10	5,158	2,251	8	0	3	0	0
			i21		(38.64)	(0.12)	(61.36)	(99.51)	(0.35)	(0)	(0.13)	(0)	(0)
**Sample 09**	YFV	9	i09	3,630	845	3	2,782	751	2	0	0	0	0
			i22		(23.28)	(0.08)	(76.72)	(99.73)	(0.27)	(0)	(0)	(0)	(0)
**Sample 10**	DENV1	10	i10	5,323	1,790	8	3,525	1,555	5	0	0	0	0
			i23		(33.63)	(0.15)	(66.37)	(99.68)	(0.32)	(0)	(0)	(0)	(0)

**Sample 11**	DENV2	11	i10 i24	7288	2,045 (28.06)	23 (0.32)	5,220 (71.94)	1,400 (98.45)	18 (1.27)	1 (0.21)	3 (0.21)	0 (0)	0 (0)
**Sample 12**	YFV	12	i12 i13	2613	791 (29.12)	0 (0)	1,822 (70.88)	657 (99.55)	0 (0.00)	0 (0)	3 (0.45)	0 (0)	0 (0)
**Average**				4659.83	1,420.75 (29.46)	6.08 (0.12)	3,233 (70.54)	1,102.17 (99.33)	4.83 (0.49)	0.25 (0.03)	1.42 (0.14)	0 (0)	0 (0)

Supplementary Table S4. Target sequence obtained from nine different viruses were cloned into plasmid then subjected to RT-PCR. Low concentration of virus (10^2 to 10^3 cp/μL) were subjected to RT-PCR the sequencing. All nine viruses can be detected and differentiated using our system and the developed pipeline. DENV: Dengue Virus, JEV: Japanese Encephalitis Virus, ZIKV: Zika Virus, YFV: Yellow Fever Virus, WNV: West Nile Virus, TBEV; Tick Borne Encephalitis Virus.

virus	Log Cp/Rx	DENV1	DENV2	DENV3	DENV4	JEV	ZIKV	YFV	WNV	TBEV	Other flaviviruses	Other viruses	Others
**DENV1**	3	**2,259**	0	0	0	0	0	0	0	0	0	0	0
**DENV2**	3	0	**1,143**	0	0	0	0	0	0	0	0	0	0
**DENV3**	3	0	0	**760**	0	0	0	0	0	0	0	0	0
**DENV4**	2	0	0	0	**869**	0	0	0	0	0	0	0	0
**JEV**	3	0	0	0	0	**1,190**	0	0	0	0	0	0	0
**ZIKV**	3	0	0	0	0	0	**947**	0	0	0	0	0	0
**YFV**	3	0	0	0	0	0	0	**2,110**	0	0	0	0	0
**WNV**	3	0	0	0	0	0	0	0	**1,270**	0	0	0	0
**TBEV**	3	0	0	0	0	0	0	0	0	**1,303**	0	0	0

Supplementary Table S5. The MinION deep sequencing was able to retrieve sequence in invisible amplicons with lower concentration. DENV: Dengue Virus, JEV: Japanese Encephalitis Virus, ZIKV: Zika Virus, YFV: Yellow Fever Virus, WNV: West Nile Virus, TBEV; Tick Borne Encephalitis Virus.

**ID**	**virus**	**Log Cp/Rx**	**DENV1**	**DENV2**	**DENV3**	**DENV4**	**JEV**	**TBEV**	**WNV**	**YFV**	**ZIKV**	**Other flaviviruses**	**Other viruses**	**Others**
1	**DENV1**	2	0	0	0	0	0	0	0	0	0	0	0	0
2	**DENV2**	2	0	0	0	0	0	0	0	0	0	0	0	0
3	**DENV3**	2	0	0	**61**	0	0	0	0	0	0	0	0	0
4	**DENV4**	1	0	0	0	**26**	0	0	0	0	0	0	0	0
5	**JEV**	1	0	0	0	0	0	0	0	0	0	0	0	0
6	**TBEV**	2	0	0	0	0	0	0	0	0	0	0	0	0
7	**WNV**	2	0	0	0	0	0	0	0	0	0	0	0	0
8	**YFV**	1	0	0	0	0	0	0	0	0	0	0	0	0
9	**ZIKV**	2	0	0	0	0	0	0	0	0	0	0	0	0

Supplementary Table S6. Distribution of deindexed reads obtained from Vietnam sample, sequenced with Flongle (top) and MinION (bottom).

	Flongle											
	i13	i14	i15	i16	i17	i18	i19	i20	i21	i22	i23	i24
i01	5	6	8	3	20	2	7	82	52	3	8	0
i02	13	2	5	2	5	2	2	4	27	5	0	3
i03	2	32	8	0	4	3	21	4	31	4	6	2
i04	3	2	2	8	5	0	24	5	18	1	0	1
i05	2	9	4	5	15	2	2	8	23	2	2	0
i06	0	1	1	2	4	0	211	0	26	2	47	4
i07	10	24	9	8	6	21	117	157	33	28	52	28
i08	7	8	0	5	4	1	17	4	10	7	3	3
i09	4	5	3	2	5	1	3	4	19	5	1	1
i10	4	3	1	8	2	2	6	2	48	5	2	0
i11	480	11	1	355	20	8	5	8	14	1	2	1
i12	2	0	1	3	4	1	74	434	11	134	34	0

	MinION											
	i13	i14	i15	i16	i17	i18	i19	i20	i21	i22	i23	i24
i01	349	515	481	251	2,111	133	312	184	3,498	402	771	446
i02	358	117	442	87	398	69	79	154	1,979	513	115	185
i03	164	2,846	765	129	161	174	961	252	1,206	236	356	180
i04	107	109	82	533	138	22	961	124	935	213	71	86
i05	72	302	57	180	276	110	66	521	760	104	66	101
i06	55	163	168	95	180	29	38	354	1,115	261	21	136
i07	474	3,287	501	762	245	1,092	5,797	19,501	2,361	2,138	3,005	3,477
i08	320	603	45	474	355	72	584	217	593	318	128	162
i09	101	195	120	164	562	36	77	195	841	112	75	109
i10	107	238	119	341	174	142	76	44	143	37	23	45
i11	0	0	0	0	1	0	0	0	0	0	0	0
i12	0	0	0	0	0	0	0	1	0	0	0	0

- **Positive** for NS1 **but negative** for DENV1-4 qPCR.
- **Positive** for **both** NS1 and DENV1-4 qPCR.
- **Negative** for NS1 and were **not** subjected to DENV1-4 qPCR.
- Sample set from experiment not included in this study.
- Unused index combination.

Supplementary Table S7. Distribution of viral reads obtained from Vietnam sample, sequenced with Flongle (top) and MinION (bottom).

Flongle	i13	i14	i15	i16	i17	i18	i19	i20	i21	i22	i23	i24
i01	**2**	0	0	0	0	0	**3**	**47**	0	0	0	0
i02	0	0	0	0	0	0	0	**2**	0	0	0	0
i03	**1**	0	0	0	0	0	0	**1**	0	0	0	0
i04	0	0	0	0	0	0	0	**1**	0	0	0	0
i05	0	0	0	0	0	0	**1**	**1**	0	0	0	0
i06	0	0	**0**	**1**	**1**	0	**179**	0	**3**	0	**43**	**1**
i07	0	0	0	0	0	0	0	**2**	0	0	0	0
i08	**1**	0	0	0	0	0	0	0	0	0	**1**	0
i09	**1**	0	0	0	0	0	**1**	**2**	0	0	0	0
i10	**1**	0	0	0	0	0	**1**	0	0	0	0	0
i11	**197**	0	0	**272**	0	**6**	**2**	**5**	**5**	**1**	0	0
i12	0	0	**1**	**1**	0	0	**59**	**370**	**2**	**1**	0	0

MinION	i13	i14	i15	i16	i17	i18	i19	i20	i21	i22	i23	i24
i01	0	0	0	0	0	0	0	0	0	0	0	0
i02	0	0	0	0	0	0	0	**1**	0	0	0	0
i03	0	0	**12**	0	0	0	0	**1**	0	0	0	0
i04	0	0	0	0	0	0	0	0	0	0	0	0
i05	0	0	0	0	0	0	0	0	0	**2**	0	0
i06	**1**	0	**16**	0	**2**	0	0	**187**	**1**	**107**	0	0
i07	0	0	0	0	0	0	0	**1**	0	0	0	0
i08	0	0	0	0	0	0	0	**60**	0	0	0	0
i09	0	0	0	0	0	0	0	0	0	**1**	0	0
i10	**2**	0	0	**1**	0	0	0	0	0	**1**	0	**1**
i11	0	0	0	0	0	0	0	0	0	0	0	0
i12	0	0	0	0	0	0	0	0	0	0	0	0

Positive for NS1 **but negative** for DENV1-4 qPCR.

Positive for **both** NS1 and DENV1-4 qPCR.

Negative for NS1 and were **not** subjected to DENV1-4 qPCR.

Sample set from experiment not included in this study.

Unused index combination.

Supplementary Table S8. Distribution of viral reads obtained from Vietnam sample, sequenced with Flongle.

DENV1	i13	i14	i15	i16	i17	i18	i19	i20	i21	i22	i23	i24
i01	0	0	0	0	0	0	3	3	0	0	0	0
i02	0	0	0	0	0	0	0	2	0	0	0	0
i03	0	0	0	0	0	0	0	1	0	0	0	0
i04	0	0	0	0	0	0	0	1	0	0	0	0
i05	0	0	0	0	0	0	1	1	0	0	0	0
i06	0	0	0	1	1	0	179	0	3	0	43	1
i07	0	0	0	0	0	0	0	1	0	0	0	0
i08	0	0	0	0	0	0	0	0	0	0	1	0
i09	0	0	0	0	0	0	1	2	0	0	0	0
i10	0	0	0	0	0	0	1	0	0	0	0	0
i11	1	0	0	272	0	6	1	4	2	0	0	0
i12	0	0	1	1	0	0	50	370	2	1	0	0

DENV2	i13	i14	i15	i16	i17	i18	i19	i20	i21	i22	i23	i24
i01	2	0	0	0	0	0	0	44	0	0	0	0
i02	0	0	0	0	0	0	0	0	0	0	0	0
i03	1	0	0	0	0	0	0	0	0	0	0	0
i04	0	0	0	0	0	0	0	0	0	0	0	0
i05	0	0	0	0	0	0	0	0	0	0	0	0
i06	0	0	0	0	0	0	0	0	0	0	0	0
i07	0	0	0	0	0	0	0	1	0	0	0	0
i08	1	0	0	0	0	0	0	0	0	0	0	0
i09	1	0	0	0	0	0	0	0	0	0	0	0
i10	1	0	0	0	0	0	0	0	0	0	0	0
i11	196	0	0	0	0	0	1	1	3	1	0	0
i12	0	0	0	0	0	0	9	0	0	0	0	0

DENV3	i13	i14	i15	i16	i17	i18	i19	i20	i21	i22	i23	i24
i01	0	0	0	0	0	0	0	0	0	0	0	0
i02	0	0	0	0	0	0	0	0	0	0	0	0
i03	0	0	0	0	0	0	0	0	0	0	0	0
i04	0	0	0	0	0	0	0	0	0	0	0	0
i05	0	0	0	0	0	0	0	0	0	0	0	0
i06	0	0	0	0	0	0	0	0	0	0	0	0
i07	0	0	0	0	0	0	0	0	0	0	0	0
i08	0	0	0	0	0	0	0	0	0	0	0	0
i09	0	0	0	0	0	0	0	0	0	0	0	0
i10	0	0	0	0	0	0	0	0	0	0	0	0
i11	0	0	0	0	0	0	0	0	0	0	0	0
i12	0	0	0	0	0	0	0	0	0	0	0	0

DENV4	i13	i14	i15	i16	i17	i18	i19	i20	i21	i22	i23	i24
i01	0	0	0	0	0	0	0	0	0	0	0	0
i02	0	0	0	0	0	0	0	0	0	0	0	0
i03	0	0	0	0	0	0	0	0	0	0	0	0
i04	0	0	0	0	0	0	0	0	0	0	0	0
i05	0	0	0	0	0	0	0	0	0	0	0	0
i06	0	0	0	0	0	0	0	0	0	0	0	0
i07	0	0	0	0	0	0	0	0	0	0	0	0
i08	0	0	0	0	0	0	0	0	0	0	0	0
i09	0	0	0	0	0	0	0	0	0	0	0	0
i10	0	0	0	0	0	0	0	0	0	0	0	0
i11	0	0	0	0	0	0	0	0	0	0	0	0
i12	0	0	0	0	0	0	0	0	0	0	0	0

(blue) **Positive** for NS1 **but negative** for DENV1-4 qPCR.
(yellow) **Positive** for **both** NS1 and DENV1-4 qPCR.
(green) **Negative** for NS1 and were **not** subjected to DENV1-4 qPCR.
(orange) Sample set from experiment not included in this study.
(grey) Unused index combination.
1 (red) Positive
1 (black) Negative

Supplementary Table S9. Distribution of viral reads obtained from Vietnam sample, sequenced with MinION (bottom).

DENV1												
	i13	i14	i15	i16	i17	i18	i19	i20	i21	i22	i23	i24
i01	0	0	0	0	0	0	0	0	0	0	0	0
i02	0	0	0	0	0	0	0	**1**	0	0	0	0
i03	0	0	0	0	0	0	0	**1**	0	0	0	0
i04	0	0	**12**	0	0	0	0	0	0	0	0	0
i05	0	0	0	0	0	0	0	0	0	2	0	0
i06	**1**	0	0	0	**2**	0	0	**187**	0	**107**	0	0
i07	0	0	0	0	0	0	0	**1**	0	0	0	0
i08	0	0	0	0	0	0	0	**60**	0	0	0	0
i09	0	0	0	0	0	0	0	0	0	**1**	0	0
i10	**2**	0	0	**1**	0	0	0	0	0	**1**	0	**1**
i11	0	0	0	0	0	0	0	0	0	0	0	0
i12	0	0	0	0	0	0	0	0	0	0	0	0

DENV2												
	i13	i14	i15	i16	i17	i18	i19	i20	i21	i22	i23	i24
i01	0	0	0	0	0	0	0	0	0	0	0	0
i02	0	0	0	0	0	0	0	0	0	0	0	0
i03	0	0	0	0	0	0	0	0	0	0	0	0
i04	0	0	0	0	0	0	0	0	0	0	0	0
i05	0	0	0	0	0	0	0	0	0	0	0	0
i06	0	0	0	0	0	0	0	0	0	0	0	0
i07	0	0	0	0	0	0	0	0	0	0	0	0
i08	0	0	0	0	0	0	0	0	0	0	0	0
i09	0	0	0	0	0	0	0	0	0	0	0	0
i10	0	0	0	0	0	0	0	0	0	0	0	0
i11	0	0	0	0	0	0	0	0	0	0	0	0
i12	0	0	0	0	0	0	0	0	0	0	0	0

DENV3												
	i13	i14	i15	i16	i17	i18	i19	i20	i21	i22	i23	i24
i01	0	0	0	0	0	0	0	0	0	0	0	0
i02	0	0	0	0	0	0	0	0	0	0	0	0
i03	0	0	0	0	0	0	0	0	0	0	0	0
i04	0	0	0	0	0	0	0	0	0	0	0	0
i05	0	0	0	0	0	0	0	0	0	0	0	0
i06	0	0	0	0	0	0	0	0	0	0	0	0
i07	0	0	0	0	0	0	0	0	0	0	0	0
i08	0	0	0	0	0	0	0	0	0	0	0	0
i09	0	0	0	0	0	0	0	0	0	0	0	0
i10	0	0	0	0	0	0	0	0	0	0	0	0
i11	0	0	0	0	0	0	0	0	0	0	0	0
i12	0	0	0	0	0	0	0	0	0	0	0	0

DENV4												
	i13	i14	i15	i16	i17	i18	i19	i20	i21	i22	i23	i24
i01	0	0	0	0	0	0	0	0	0	0	0	0
i02	0	0	0	0	0	0	0	0	0	0	0	0
i03	0	0	0	0	0	0	0	0	0	0	0	0
i04	0	0	0	0	0	0	0	0	0	0	0	0
i05	0	0	0	0	0	0	0	0	0	0	0	0
i06	0	0	**16**	0	0	0	0	**1**	0	0	0	0
i07	0	0	0	0	0	0	0	0	0	0	0	0
i08	0	0	0	0	0	0	0	0	0	0	0	0
i09	0	0	0	0	0	0	0	0	0	0	0	0
i10	0	0	0	0	0	0	0	0	0	0	0	0
i11	0	0	0	0	0	0	0	0	0	0	0	0
i12	0	0	0	0	0	0	0	0	0	0	0	0

- **Positive** for NS1 **but negative** for DENV1-4 qPCR.
- **Positive** for **both** NS1 and DENV1-4 qPCR.
- **Negative** for NS1 and were **not** subjected to DENV1-4 qPCR.
- Sample set from experiment not included in this study.
- Unused index combination.
- **1** Positive
- **1** Negative

Supplementary Table S10. Result of pan-flavivirus system in clinical sample from Brazil

Sample No	Barcode	Index F	Index R	Result of RT-qPCR*	pan-flavi-PCR**	Virus reads							
						SLEV	YFV	WNV	DENV1	DENV2	DENV3	DENV4	ZIKV
1	3	i01	i13	SLEV	++	**118,350**	**2**	**1**	0	**1**	0	**47**	0
2	3	i02	i14	SLEV	++	**62,830**	**3**	**15**	**1**	**1**	0	**28**	0
3	3	i03	i15	WNV	+	**4**	**4**	**5,591**	0	0	0	**5**	0
4	3	i04	i16	WNV	++	**2**	0	**29,035**	0	**1**	**1**	**3**	0
5	3	i05	i17	YFV	++	**5**	**23,420**	0	0	**1**	0	**11**	0
6	3	i07	i19	DENV	++	**6**	**6**	**1**	**99**	**5,006**	**1,334**	**27,848**	0
7	3	i08	i20	DENV	+	**29**	**23**	**6**	**495**	**325**	**104**	**3,336**	0
8	1	i09	i21	ZIKA	+/-	**2**	0	**3**	**749**	0	0	0	0
9	1	i10	i22	ZIKA	-	**2**	0	0	0	0	0	0	0
10	1	i11	i23	DENV4 and ZIKV	++	**30**	**1**	0	0	0	0	**2560**	0
11	1	i12	i24	YFV	+	**3**	**4,018**	0	0	**1**	0	0	0
12	2	i01	i13	ZIKA	++	**105**	0	0	0	0	0	**27**	**1217**
13	2	i02	i14	YFV	+	**63**	**32,141**	0	0	0	0	**12**	0
14	2	i06	i18	DENV4	++	0	0	0	0	0	0	**21,974**	0
15	2	i08	i20	WNV	+/-	**1**	**2**	0	0	**2**	**1**	**24**	0
16	2	i09	i21	unknown	+/-	0	**150**	0	**2**	0	0	**4**	0
17	2	i05	i17	unknown	+/-	0	**38**	0	0	0	0	0	0
18	2	i07	i19	unknown	+/-	0	0	0	0	**2**	**2**	**23**	0
19	2	i04	i16	unknown	-	0	0	**30**	0	0	0	0	0
20	2	i10	i22	unknown	-	0	**1**	0	0	0	0	**5**	0
21	2	i11	i23	unknown	-	0	**2**	0	0	0	0	**32**	0
22	2	i12	i24	unknown	-	0	**13**	0	0	0	0	0	0
23	2	i03	i15	YFV	-	0	**1**	**8**	0	0	0	**2**	0
24	1	i06	i18	YFV	-	0	0	0	**1**	0	0	**7**	0

*) Diagnostic decision by qRT-PCR. The question mark (?) represent uncertainty owing to their high Ct value.
**) Intensity of gel electrophoresis of PCR products
++: marked strong

+: strong
+/-: ambiguous
-: negative
SLEV = Saint Louis Encephalitis Virus; YFV = Yellow Fever Virus; WNV = West Nile Virus; DENV = Dengue Virus; ZIKV = Zika Virus.
